# Integration of Multiplex Bead Assays for Parasitic Diseases into a National, Population-Based Serosurvey of Women 15-39 Years of Age in Cambodia

**DOI:** 10.1371/journal.pntd.0004699

**Published:** 2016-05-03

**Authors:** Jeffrey W. Priest, M. Harley Jenks, Delynn M. Moss, Bunsoth Mao, Sokhal Buth, Kathleen Wannemuehler, Sann Chan Soeung, Naomi W. Lucchi, Venkatachalam Udhayakumar, Christopher J. Gregory, Rekol Huy, Sinuon Muth, Patrick J. Lammie

**Affiliations:** 1 Division of Foodborne, Waterborne, and Environmental Diseases at the Centers for Disease Control and Prevention, Atlanta, Georgia, United States of America; 2 Division of Parasitic Diseases and Malaria, Centers for Disease Control and Prevention, Atlanta, Georgia, United States of America; 3 University of Health Sciences, Phnom Penh, Cambodia; 4 National Institute of Public Health, Phnom Penh, Cambodia; 5 Global Immunization Division, Center for Global Health, Centers for Disease Control and Prevention, Atlanta, Georgia, United States of America; 6 National Immunization Program, Ministry of Health, Phnom Penh, Cambodia; 7 Center for Parasitology, Entomology, and Malaria Control, Phnom Penh, Cambodia; University of Edinburgh, UNITED KINGDOM

## Abstract

Collection of surveillance data is essential for monitoring and evaluation of public health programs. Integrated collection of household-based health data, now routinely carried out in many countries through demographic health surveys and multiple indicator surveys, provides critical measures of progress in health delivery. In contrast, biomarker surveys typically focus on single or related measures of malaria infection, HIV status, vaccination coverage, or immunity status for vaccine-preventable diseases (VPD). Here we describe an integrated biomarker survey based on use of a multiplex bead assay (MBA) to simultaneously measure antibody responses to multiple parasitic diseases of public health importance as part of a VPD serological survey in Cambodia. A nationally-representative cluster-based survey was used to collect serum samples from women of child-bearing age. Samples were tested by MBA for immunoglobulin G antibodies recognizing recombinant antigens from *Plasmodium falciparum* and *P*. *vivax*, *Wuchereria bancrofti*, *Toxoplasma gondii*, *Taenia solium*, and *Strongyloides stercoralis*. Serologic IgG antibody results were useful both for generating national prevalence estimates for the parasitic diseases of interest and for confirming the highly focal distributions of some of these infections. Integrated surveys offer an opportunity to systematically assess the status of multiple public health programs and measure progress toward Millennium Development Goals.

## Author Summary

In 2012 a comprehensive national serosurvey to assess immunity to vaccine preventable diseases such as polio, rubella, measles, and tetanus was conducted among women of child bearing age in Cambodia. We were able to test this sample set using a multiplex bead assay in order to measure specific antibody responses to the parasites that cause malaria, toxoplasmosis, lymphatic filariasis, cysticercosis, and strongyloidiasis. National prevalence estimates generated from the serologic data show widespread exposure (>45% positive) to the soil transmitted nematode worm, *Strongyloides stercoralis*. In contrast, <5% of women were positive for antibodies to *P*. *falciparum* malaria, *P*. *vivax* malaria, and lymphatic filariasis, and antibody-positive women were mainly found in the North region of the country. Women who were positive for antibodies to *Toxoplasma gondii* and *Taenia solium* (5.8% and 2.6% of the population, respectively) were not clustered in any particular geographic region. With this study we have shown how the integration of a multiplex assay into a national serosurvey can provide useful information on the prevalence and distributions of medically important parasitic infections.

## Introduction

In many tropical and sub-tropical countries, the disease burden represented by neglected tropical diseases (NTDs) is substantial, yet information on the prevalence and distribution of these diseases is limited because of the significant costs associated with disease-specific surveys. Even with the recent scale-up of preventive chemotherapy programs targeting NTDs [1], routine assessments to monitor the impact of these programs, when they occur, are often restricted to sentinel sites and may not be representative of all program areas. For some diseases such as strongyloidiasis, prevalence data for many regions of the world are lacking, and no public health strategy has been developed for control of the disease [2, 3].

Demographic and Health Surveys (DHS) and other population-based multiple indicator surveys are conducted to assess the performance of health and development programs. The United States Agency for International Development (USAID) has assisted in over 230 DHS surveys in more than eighty countries since 1984 at a cost of approximately $380 million dollars, and additional monies have been contributed by other donors as well as host countries [4]. Collection of biomarker data is often included in these types of population-based surveys to assess morbidity, HIV status, or malaria infection prevalence, but these surveys have not been extended to include NTDs. Multiplexing technologies provide new opportunities to collect data on a large number of diseases using a single serum sample or dried blood spot [5]. Such an approach would provide Ministries of Health with valuable information on the distribution and prevalence of NTDs, opportunities to monitor the impact of NTD interventions, evidence to inform programmatic decisions, and post-elimination surveillance.

The Cambodian Ministry of Health conducted a serological survey in 2012 to assess population immunity for poliomyelitis, measles, rubella and tetanus among women aged 15–39 years [6]. This comprehensive national serological survey provided an excellent opportunity to gather information on the distribution and prevalence of other diseases throughout Cambodia by measuring antibody responses to a panel of antigens representing several parasitic infections. We used multiplexing technology to assay sera collected in this national serological survey for immunoglobulin G (IgG) antibodies against tetanus, measles, *Plasmodium falciparum* and *P*. *vivax*, *Wuchereria bancrofti*, *Toxoplasma gondii*, *Taenia solium*, *Strongyloides stercoralis*, and several arthropod-borne viruses. For *S*. *stercoralis* the national prevalence exceeded 40% and was indicative of a country-wide public health problem of surprising magnitude. Multiplexed approaches provide an opportunity to gather information of public health importance on a large scale using well-standardized survey platforms and well-characterized infection markers.

## Materials and Methods

### Survey design

Samples were obtained during a serological survey in November and December 2012 as previously described [6]. Briefly, blood samples were collected from women of child-bearing age (15–39 years) throughout Cambodia. Multi-stage cluster sampling was performed with oversampling of areas identified as higher risk for tetanus based on the 2009 Cambodian neonatal tetanus risk assessment. One hundred enumeration areas (EAs) were selected by simple random sampling of the 611 EAs defined for Cambodia’s 2010 DHS survey. The number of rural and urban EAs from each region were selected to match the relative proportion of urban and rural populations in the region. From each of the EAs, twenty-two households were selected and all eligible women in those households were invited to participate. The design and sample size were selected to provide estimates of population rubella and tetanus immunity nationwide and by age-group [6, 7].

Five milliliters of whole blood were collected from each participant, and sera were separated shortly thereafter and stored at -80°C. As previously described, samples were initially tested by enzyme-linked immunosorbent assay (ELISA) or standard microneutralization assay for antibodies to measles, rubella, and polio [6]. Residual samples were then tested by multiplex bead assay (MBA) at CDC in Atlanta, GA, and by double antigen ELISA for tetanus antibody levels at the Statens Serum Institut, Copenhagen, Denmark. The results of measles, rubella and polio antibody testing have been published [3] and the tetanus assay results will be reported elsewhere [7]. As previously described [7], a total of 2150 samples had reported tetanus values and were included in the multiplex assay testing.

### Ethics statement

Written informed consent was obtained and documented prior to participation in the survey; specific consent for serologic testing of diseases of public health importance was included as part of this process. Consent was also be obtained separately from the parent or guardian of women under the age of 18. The protocol was reviewed and approved by the national ethics committee in Cambodia.

### Recombinant parasite antigens used

Staff of the Ministry of Health of Cambodia selected antigens to be included in the multiplex. The following parasite-specific recombinant antigens were used in the MBA (Table 1): NIE for *Strongyloides stercoralis* [8]; SAG2A for *Toxoplasma gondii* [9, 10]; T24H for cysticercosis [11]; PfMSP-1_19_ (3D7 strain) and PfMSP-1_42_ (3D7 strain and FVO strain) for *P*. *falciparum* malaria [12, 13]; and PvMSP-1_19_ (Belem strain) for *P*. *vivax* malaria [14, 15]. For lymphatic filariasis, *Brugia malayi* Bm14 (SXP-1) [16], *B*. *malayi* Bm33 (Bm-AP-1) [17], and *W*. *bancrofti* Wb123 [18] antigens were used. Wb123 is reported to be largely species specific [18, 19], while the Bm14 and Bm33 antigens cross react with sera from *W*. *bancrofti* infected patients as well as with sera from some patients infected with other filarial worm species [17, 20].

**Table 1:** Coupling conditions and cutoff values for antigens used in parasitic disease MBA.

Infection (strain)	Antigen	Tag^a^	Protein (μg)^b^	pH^c^	Cutoff (MFI-bg)
Lymphatic filariasis	Bm14	GST	120	7.2	65
Lymphatic filariasis	Wb123	GST	120	7.2	115
Lymphatic filariasis	Bm33	GST/His	20	6.0^d^	966
Strongyloidiasis	NIE	GST	20	7.2^d^	792
Toxoplasmosis	SAG2A	GST	20^e^	5.0	159
Cysticercosis	T24H	GST	120	5.0	486
Malaria (3D7)^f^	PfMSP1_19_	GST	30	5.0	343
Malaria (3D7)^f^	PfMSP1_42_	None	15	5.0	295
Malaria (FVO)^f^	PfMSP1_42_	None	15	5.0	141
Malaria (Belem)^g^	PvMSP1_19_	GST	20	5.0	196
Negative control	None^h^	GST	15	5.0	None

^***a***^GST, *S*. *japonicum* glutathione-*S*-transferase; His, histidine_6_.

^***b***^Protein amount used in a 0.5 ml coupling reaction with 12.5 x 10^6^ SeroMap beads.

^***c***^pH 5.0 and 6.0 couplings conducted in MES/ NaCl buffer. pH 7.2 couplings conducted in Na_2_HPO_4_/ NaCl buffer. See text for concentrations.

^***d***^Coupling buffer included 2 M urea.

^***e***^Note that later studies decreased SAG2A to 12.5 μg/ml of beads [22].

^**f**^P. falciparum.

^**g**^P. vivax.

^***h***^Recombinant GST in the absence of additional protein sequence was coupled to beads as a negative control.

Recombinant Bm14 [21], SAG2A [22], and NIE [23] proteins tagged with *Schistosoma japonicum* glutathione-*S*-transferase (GST) and control GST with no fusion partner [24] were expressed and purified as described elsewhere. Bm33 [25] and T24H [26] were expressed with GST fused to the amino terminus and with six histidines (His_6_) on the carboxy terminus and purified as previously described. Following purification, the His_6_ tag was removed from T24H by Factor Xa cleavage [26]. Recombinant PfMSP-1_19_-GST (3D7 parasite strain) fusion protein and PfMSP-1_42_ proteins (3D7 and FVO parasite strains) lacking fusion tags were provided by C. Kauth and H. Bujard (Heidelberg University, Heidelberg, Germany) [27]. Wb123-GST fusion protein was provided by Dr. T. Nutman (NIH, Bethesda, MD).

The *P*. *vivax* PvMSP1_19_-GST was cloned, expressed, and purified for the MBA. The coding sequence (including the carboxy-terminal, hydrophobic anchor sequence) was amplified from Belem strain DNA (provided by J. Barnwell, CDC, Atlanta, GA) using the following forward and reverse deoxyoligonucleotide PCR primers: 5’-CGC GGA TCC ACT ATG AGC TCC GAG CAC ACA TG-3’ and 5’-GCG GAA TTC TTA AAG CTC CAT GCA CAG GAG-3’, respectively. *Bam*HI and *Eco*RI restriction endonuclease sites used for directional cloning into pGEX 4T-2 plasmid (GE Healthcare, USA) are underlined in the primer sequences. Polymerase chain reaction amplification conditions and protocols for cloning into *Escherichia coli* BL21 cells (Stratagene, USA) have previously been described [28]. The sequence of the resulting PvMSP1_19_ clone was confirmed to match that found in GenBank (accession number AF435594.1) [29]. Recombinant PvMSP1_19_-GST fusion protein was expressed and purified on a glutathione Sepharose 4B affinity column as directed by the manufacturer (GE Healthcare). Glutathione-eluted proteins were dialyzed overnight against 300 volumes of 25 mM Tris buffer at pH 7.5 using Spectra-Por3 dialysis membrane (3,500-Da cutoff, Spectrum Laboratories, Rancho Dominguez, CA). Proteins were bound to a Mono Q HR5/5 strong anion exchange column (GE Healthcare) and eluted with a 20 min linear gradient from 0 to 0.25 M NaCl in 25 mM Tris buffer at pH 7.5. Protein fractions collected between 0.15 and 0.21 M NaCl were mostly free of contaminants by SDS polyacrylamide gel analysis and were combined. The final protein product was dialyzed against 300 volumes of PBS and then concentrated to approximately 1 mg/ml using a Centricon-10 centrifugal filter device (Millipore Corporation, Bedford, MA). The yield from 2 L of *E*. *coli* cells was approximately 1.5 mg of purified PvMSP1_19_-GST protein.

### Multiplex bead antibody assays for parasitic diseases

Bm14-GST and Wb123-GST antigens (120 μg for 12.5 x 10^6^ beads in 0.5 ml) were covalently coupled to SeroMap microsphere beads (Luminex Corp., Austin, TX) using conditions previously described in buffer containing 10 mM Na_2_HPO_4_ and 0.85% NaCl at pH 7.2 (PBS) [25]. For the other antigens, coupling buffers for conjugation were empirically chosen to minimize protein usage and maximize the signal/ noise ratio (shown in Table 1). A small scale coupling reaction (50 μl containing 6.25 x 10^5^ beads) conducted at a protein concentration of 120 μg/ ml in PBS at pH 7.2 was compared to small scale coupling reactions performed in phosphate buffer at pH 7.2 or in buffers containing 2-(*N*-morpholino)-ethanesulfonic acid (MES) at pH 5.0 or 6.0. Protein concentrations were varied from 120 μg/ ml to as low as 10 μg/ ml. Each small scale coupling was conducted using a different bead classification number so that the beads could be combined in a single assay well for analysis. The efficiencies of the couplings were compared by MBA (conditions described below) using a serial dilution of a strong positive human serum, a panel of positive and negative human sera, or a 10-fold serial dilution of a goat anti-GST polyclonal antibody (GE Healthcare) with a biotinylated rabbit anti-goat IgG secondary antibody (1:500 dilution; Invitrogen, Carlsbad, CA). Antigens coupled in 0.5 ml of 25 mM MES at pH 5.0 with 0.85% NaCl used the following amounts of protein for 12.5 x 10^6^ beads: SAG2A-GST, 20 μg; T24H-GST, 120 μg; PfMSP-1_19_-GST, 30 μg; PfMSP-1_42_ proteins, 15 μg; PvMSP1_19_-GST, 20 μg; GST control protein, 15 μg. The two antigens purified in the presence of 2 M urea required 2 M urea in the coupling buffer to minimize the MBA response to negative human sera. Bm33-GST-His_6_ (20 μg for 12.5 x 10^6^ beads in 0.5 ml) was coupled in buffer containing 25 mM MES, 2 M urea, and 200 mM NaCl at pH 6.0. NIE-GST (20 μg for 12.5 x 10^6^ beads in 0.5 ml) was coupled in buffer containing 50 mM Na_2_HPO_4_, 2 M urea, and 200 mM NaCl at pH 7.2.

Test sera were diluted 1:400 in PBS buffer (pH 7.2) containing 0.3% Tween-20, 0.02% sodium azide, 0.5% BSA, 0.5% casein, 0.5% polyvinyl alcohol (PVA), 0.8% polyvinylpyrrolidone (PVP), and 3 μg/ml *E*. *coli* extract, and duplicate samples were assayed for total IgG antibodies as previously described [21, 25, 30]. Casein was found to provide additional background noise reductions for the NIE and Bm33 assays compared to PVA and PVP alone. Assay performance was monitored by the inclusion on each plate of two positive control serum dilutions, two negative control serum dilutions, and a buffer-only blank. The average of the median fluorescent intensity values from the duplicate wells *minus* the background fluorescence from the buffer-only blank was reported as the “median fluorescence intensity *minus* background” (MFI-bg). Samples having a coefficient of variation of >15% for ≥2 positive responses between the duplicate wells were repeated.

Because several of the parasitic diseases represented in our MBA panel are not endemic in the United States (U.S.), we were able to use serum samples from 86 healthy, adult US citizens with no history of foreign travel to define positive IgG response cutoffs [25]. Cutoffs for Bm14 (65 MFI-bg), Wb123 (115 MFI-bg), Bm33 (966 MFI-bg), NIE (792 MFI-bg), and T24H (486 MFI-bg) were based on the mean plus three standard deviations of the respective antibody response values (Table 1). Cutoffs for PfMSP1_19_ (343 MFI-bg) and PvMSP1_19_ (196 MFI-bg) were calculated using the mean plus three standard deviations of log transformed antibody response values (Table 1) [31]. Panels of parasitologically confirmed, anonymous sera were available for MBA sensitivity determinations for malaria antigens (slide microscopy positive patients, *P*. *falciparum n* = 33 and *P*. *vivax n* = 35), *S*. *stercoralis* NIE antigen (stool positive patients, *n* = 44), and cysticercosis T24H antigen (patients with multiple cysts confirmed by CT or MRI scan, *n* = 52).

The significant prevalence of toxoplasmosis in the U.S. population [32] required the use of an alternate means of cutoff determination for the SAG2A antigen. A panel of positive and negative sera (*n* = 45) identified using the “gold standard” Sabin-Feldman dye binding assay was tested by MBA, and the average of the highest negative value (22 MFI-bg) and the lowest positive value (295 MFI-bg) was chosen as the positive cutoff (159 MFI-bg) (Table 1) [22, 33].

### Statistical analysis

An alpha of 0.05 was set for tests of statistical significance. Statistical analyses were conducted using SAS v9.3 (Cary, NC, USA) and STATA v 13.1 (College Station, TX, USA). Briefly, sampling weights were calculated to take each stage of selection into account, including the probability of selecting the original EAs in the 2010 DHS. A non-response adjustment by strata was included using the weighting class approach. Final weights were scaled to conform to the regional distribution of the population in the 2008 census [34]. Estimates of seroprevalence and coverage with 95% (logit) confidence intervals (CI) were calculated accounting for survey design (STATA v13.1). Second-order Rao-Scott Chi-square tests were used to assess differences in seroprevalence across age groups, regions, and rural/urban residence.

## Results

The cutoff values assigned to the various parasite MBAs in Table 1 performed well when the assays were used to test serum panels from parasite infection-confirmed patients. All three *P*. *falciparum* MBAs detected IgG antibodies in 75.8% of the slide positive serum panel, and each positive serum reacted with all three antigens. Unfortunately, demographic information and details on the timing of sample collection relative to malaria infection were not available for this anonymous sample set. Specificities calculated from the U.S. citizen negative control panel ranged from 100% (PfMSP1_19_) to 96.5% (PfMSP1_42_). The PvMSP1_19_, *S*. *stercoralis* NIE, and cysticercosis T24H MBAs had sensitivities of 85.7%, 84.1%, and 98.1%, respectively. Specificities calculated from the U.S. citizen negative control panel were 98.8% for each assay. As previously reported, the *T*. *gondii* SAG2A MBA was 100% sensitive and specific compared to a “gold standard” assay defined panel [22]. Sensitivities were not determined for the LF antigen MBAs; they were >97.7% specific with our U.S. negative samples.

**Fig 1:**
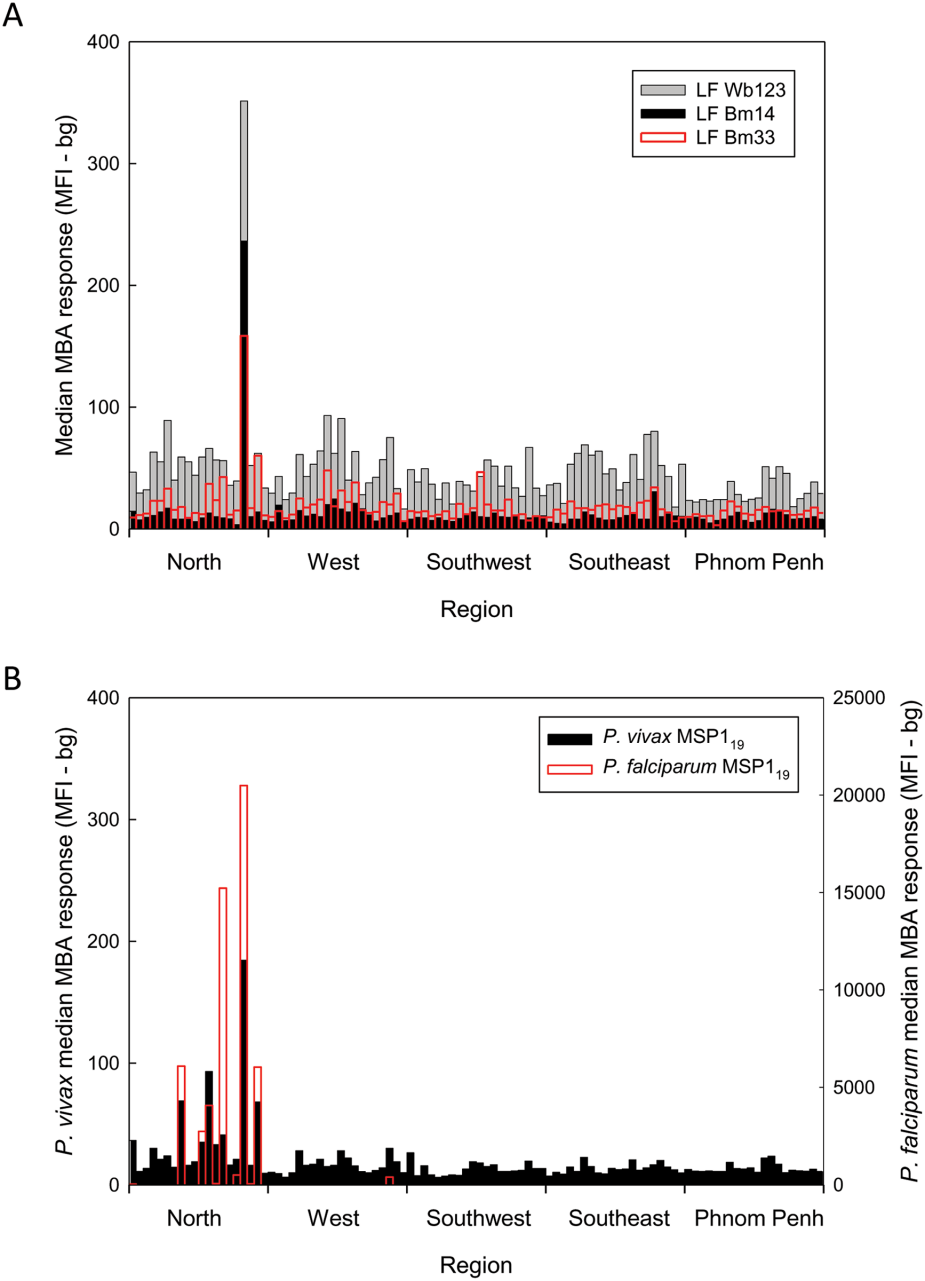
Median plots for LF responses (A) and malaria responses (B) in Cambodian women 15–39 years of age. A, Multiplex bead assay results for Bm14 (black), Bm33 (red), and Wb123 (gray) were grouped first by Enumeration Area (EA) and then by geographic region as follows: North (Banteay Mean Chey, Kampong Thom, Kratie, Mondolkiri, Otdar Mean Chey, Preah Vihear, Ratanakiri, Siem Reap, and Steung Treng provinces), West (Battambang, Kampong Chhang, Kampong Speu, Koh Kong, Pailin, Preah Sihanouk, and Pursat provinces), Southwest (Kampot, Kandal, Kep, and Takeo provinces), Southeast (Kampong Cham, Prey Veng, and Svay Rieng provinces), and Phnom Penh [6]. A median MFI-bg result was calculated for each EA and is plotted versus region. A single coincident peak of LF reactivity was noted in a single EA in the North region. B, Median multiplex results for *P*. *vivax* (black) and *P*. *falciparum* (red) MSP1_19_ antigens were calculated as described in A. Note that the results for *P*. *vivax* MSP1_19_ are plotted on the left hand *y*-axis while those for *P*. *falciparum* MSP1_19_ are plotted on the right hand *y*-axis.

**Fig 2:**
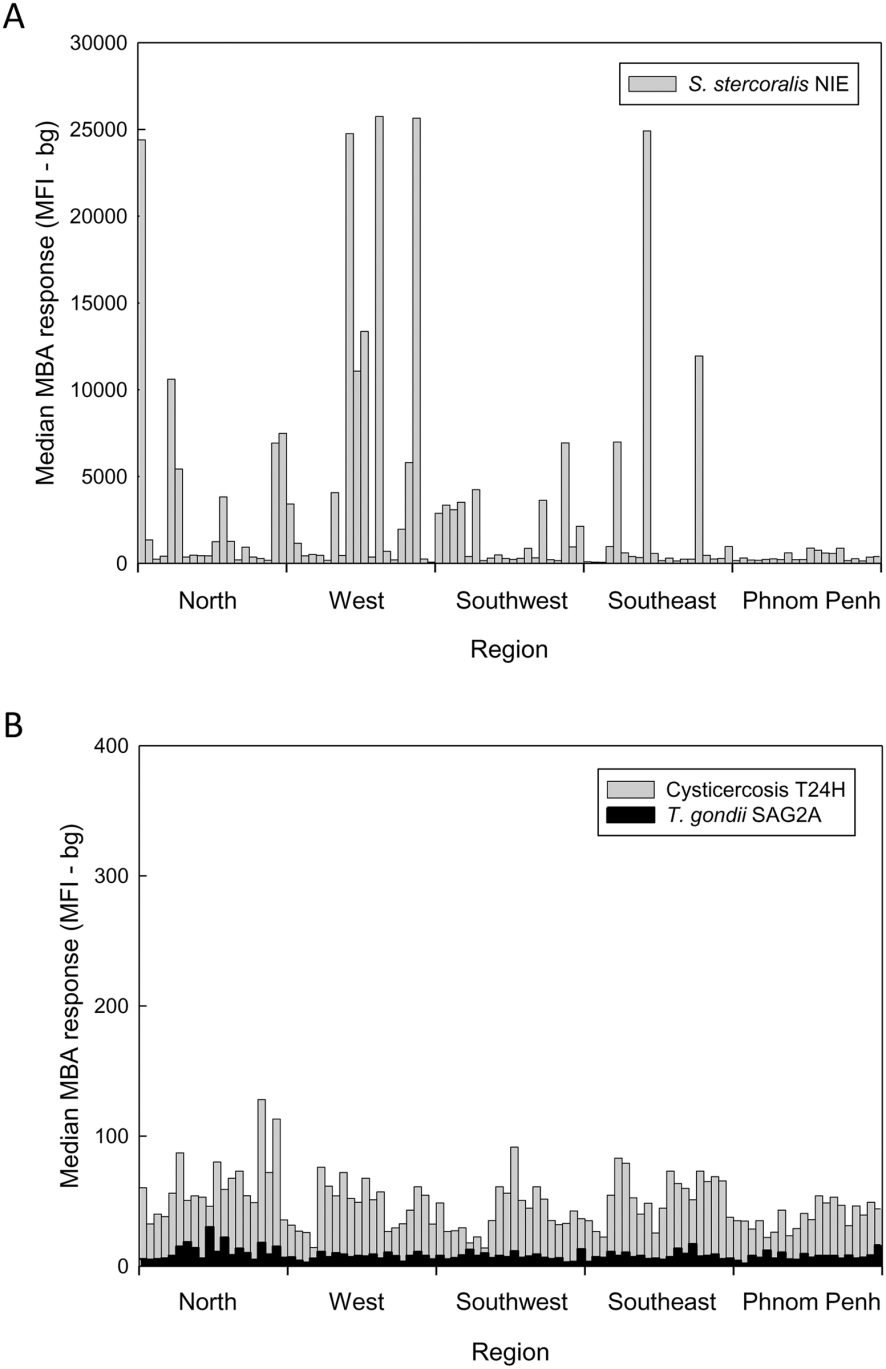
Median plots for parasitic disease responses in Cambodian women 15–39 years of age. Antibody results for a strongyloidiasis antigen (A), and toxoplasmosis (black) and cysticercosis (gray) antigens (B) were grouped as described in Fig 1. A median MFI-bg result was calculated for each EA and is plotted versus region.

Although the Cambodia population survey was not powered to detect differences in antibody prevalence across EAs, a plot of median values of the data sorted first by EA and then by region revealed distinct high and low median prevalence values for some of the parasite-specific antibody responses. A single EA in the Steung Treng province of the North geographic region of the country was found to have coincident peaks of high antibody responses to all three LF antigens (Fig 1A). Multiple North region EAs located in the provinces of Kratie, Preah Vihear, Ratanakiti, and Steung Treng had coincident peaks of antibody reactivity to the *P*. *falciparum* and the *P*. *vivax* MSP1_19_ antigens (Fig 1B). A weak median response peak in the West region province of Pursat was also detected. Median antibody response plots for the FVO and 3D7 PfMSP1_42_ antigens largely mirrored those observed with the PfMSP1_19_ antigen (S1 Fig). Because the MSP1_42_ antigens included the MSP1_19_ sequence, these responses were not further analyzed. Multiple peaks of antibody to *S*. *stercoralis* NIE were observed throughout the country, and only the largely urban Phnom Penh region had EAs with relatively low median responses (Fig 2A). For toxoplasmosis and cysticercosis there was no discernible geographic clustering of the antibody responses detected (Fig 2B).

Weighted national estimates for toxoplasmosis and cysticercosis were 5.8% (CI, 4.7–7.0) and 2.6% (CI, 1.8–3.7), respectively, with no statistically significant urban/ rural, regional, or age-related differences noted (S1 Table). Weighted national estimates of seroprevalence for *P*. *falciparum* (4.6%; CI, 3.1–6.8) and *P*. *vivax* (4.6%; CI, 3.3–6.4) malaria are shown in Table 2. Antibody prevalence was significantly higher in rural areas than urban areas for *P*. *falciparum (P* = 0.005*)* and *P*. *vivax (P* = 0.014*)*. Regional differences in seroprevalence were statistically significant for *P*. *falciparum and P*. *vivax* (Table 2), with the North region having higher seroprevalence than the other regions combined for *P*. *falciparum* (13.7% vs. 1.9%; P < 0.001) and *P*. *vivax (9*.*2% vs*. *3*.*2%; P* = 0.003*)*. No age related differences were noted for either malaria species. For the LF estimate (Table 3) we required that antibodies to two or more of the LF antigens be present for a sample to be considered positive. The national LF estimate was low at only 2.4% (CI, 1.6–3.6), but statistically significant rural/ urban (*P* = 0.039) and regional (*P* < 0.001) differences were observed with the latter driven by a higher prevalence of 5.6% (CI, 3.0–10.2) in the North region. In contrast to the low seroprevalence estimates for the parasitic diseases mentioned above, just under half of women of child-bearing age in our countrywide sample of Cambodia were positive for antibodies to *S*. *stercoralis* (45.9%, CI, 41.7–50.1) (Table 3). Differences between regions (P < 0.001) and between urban and rural populations were highly significant (*P* = 0.003), but no age differences were detected (*P* = 0.195).

**Table 2:** 

		*P*. *falciparum* MSP1_19_ MBA^a^					*P*. *vivax* MSP1_19_ MBA^a^				
Characteristic	Total	Positive	Percent	LCL	UCL	*P* value	Positive	Percent	LCL	UCL	*P* value
Overall	2150	174	4.6	3.1	6.8		114	4.6	3.3	6.4	
Residence type											
Urban	655	14	2.0	1.1	3.6	**0.005**	16	2.1	1.1	4.2	**0.014**
Rural	1495	160	5.3	3.4	8.2		98	5.3	3.7	7.6	
Region											
North	394	131	13.7	6.8	25.7	**<0.001**	56	9.2	4.7	17.5	**0.003**
West	445	24	3.3	1.0	10.2		22	4.1	2.2	7.6	
Southwest	423	7	1.1	0.5	2.8		17	4.2	2.1	8.1	
Southeast	419	5	1.4	0.7	3.1		9	2.1	1.1	4.2	
Phnom Penh	469	7	1.2	0.5	2.7		10	1.9	0.8	4.7	
Age group (yr)											
15–19	435	34	3.9	1.9	7.9	0.245	20	3.0	1.8	5.1	0.249
20–24	468	34	4.0	2.5	6.3		22	3.6	2.0	6.2	
25–29	483	32	4.3	2.5	7.2		26	3.9	2.4	6.0	
30–34	449	35	4.2	2.4	7.2		22	5.9	2.2	14.6	
35–39	315	39	6.9	4.1	11.4		24	7.0	4.9	10.0	

^a^Estimates adjusted to account for sampling weights and survey design.

Abbreviations: LCL, Lower confidence limit; UCL, Upper confidence limit.

**Table 3:** 

		*Strongyloides stercoralis* NIE MBA^a^					*Lymphatic filariasis* MBA (any two antigens)^a^				
Characteristic	Total	Positive	Percent	LCL	UCL	*P* value	Positive	Percent	LCL	UCL	*P* value
Overall	2150	935	45.9	41.7	50.1		52	2.4	1.6	3.6	
Residence type											
Urban	655	197	32.4	23.4	43.0	**0.003**	9	1.0	0.4	2.7	**0.039**
Rural	1495	738	49.8	44.9	54.6		43	2.8	1.9	4.3	
Region											
North	394	202	58.3	47.1	68.6	**<0.001**	26	5.6	3.0	10.2	**<0.001**
West	445	234	52.7	42.7	62.4		7	1.4	0.6	3.1	
Southwest	423	192	42.9	35.5	50.7		7	1.8	0.9	3.5	
Southeast	419	167	39.0	30.6	48.1		5	1.4	0.6	3.6	
Phnom Penh	469	140	26.1	19.2	34.3		7	1.2	0.5	3.2	
Age group (yr)											
15–19	435	160	39.9	33.7	46.5	0.195	9	1.5	0.8	3.0	0.323
20–24	468	196	45.4	37.9	53.0		11	1.7	0.5	5.5	
25–29	483	211	43.8	37.0	50.8		13	2.5	1.3	4.7	
30–34	449	206	51.0	44.7	57.2		11	2.4	1.0	5.9	
35–39	315	162	49.8	39.8	59.9		8	4.3	2.3	7.7	

^a^Estimates adjusted to account for sampling weights and survey design.

Abbreviations: LCL, Lower confidence limit; UCL, Upper confidence limit.

## Discussion

In a previous report, we used the multiplex bead assay to determine anti-tetanus toxoid antibody levels in Cambodian women of child-bearing age and demonstrated that the estimates of population immunity derived from the multiplex testing were very similar to those derived from the “gold standard” assay methodologies [7]. Here we demonstrate that multiplexed antibody assays, when integrated into the robust, population-based Cambodian serologic survey framework, can be used to provide nationally-representative estimates of the presence and distribution of a number of parasitic diseases of public health importance. Although others have used multiplex assays to measure multiple anti-parasite antibody responses [35], this report is, to our knowledge, the first to generate national parasitic disease estimates from multiplexed serologic antibody assays.

Cambodia recently completed five years of mass drug administration (MDA) to eliminate lymphatic filariasis in a small number of northern and northeastern provinces where the presence of infection had been documented by antigen surveys (http://www.who.int/neglected_diseases/preventive_chemotherapy/lf/en/) [36]. Our results demonstrate the presence of significant residual antibody reactivity in the geographic North area where the MDA occurred and, perhaps of greater importance, its relative absence in areas where MDA was not carried out. These results are an important confirmation of the baseline mapping data that was used as the basis for determining where to implement MDA. The presence of residual antibody following MDA, as high as 60% in one EA, is not surprising as antifilarial antibody responses in adults are known to be long-lived [37, 38]. Although sampling of children may be of greater value in the post-MDA setting as a measure of incident seroconversions, these results suggest the potential use of LF antibody testing as a tool for LF surveillance. Additional information on the longevity of responses in adults is needed to guide recommendations on the use of antibody surveys for post-MDA surveillance.

The two other vector borne parasitic infections in our panel, *P*. *falciparum* and *P*. *vivax* malaria, were also focally distributed with seroprevalence for PfMSP1_19_ antibody approaching 100% in some EAs (S1 Data). Both of our national malaria seroprevalence estimates (4.6% for *P*. *falciparum* and 4.6% for *P*. *vivax*) were considerably higher than the 0.9% blood film parasite prevalence estimate for all species of malaria generated by the Cambodia Ministry of Health in 2010 [39]. From samples collected in Cambodia in 2005, Cook et al. [40] reported a *P*. *falciparum* peak seasonal seroprevalence of 49.2% compared to a parasite prevalence by slide microscopy of only 3.4% (November, western provinces) and a *P*. *vivax* seroprevalence of 20.2% compared to a 10.7% parasite prevalence (August, eastern province). The discrepancies between the parasite prevalence by blood film microscopy and the parasite-specific IgG antibody prevalence may reflect low malaria parasite loads that remain below the limit of microscopic detection [41, 42], or, as in the case of LF described above, may result from a long IgG titer half-life following successful treatment [43]. Although the public health value of malaria serosurveys in adults may be limited to confirming the known distributions of those infections, serosurveys in young children, as with LF, may provide useful surveillance data for mapping transmission foci in the context of malaria elimination efforts and may offer an opportunity to monitor the impact of interventions by documenting reductions in seroprevalence over time [44, 45].

From our MBA results, prevalence of IgG antibody to toxoplasmosis (3.5–7.3%) and to cysticercosis (1.3–3.3%) was relatively low across all geographic regions. Few surveys have been conducted for either of these infections in Cambodia [46, 47], but our values are consistent with the limited information available. Seroprevalence of IgG antibodies to *T*. *gondii* among women <40 years of age in Phnom Penh was 8.4% in one small study [48]. A low seroprevalence suggests that the majority of women of child-bearing age in Cambodia are at risk of primary *T*. *gondii* infection and could, if infected during pregnancy, transmit toxoplasmosis to their babies *in utero* with serious health consequences [49, 50].

Recent stool-based detection surveys by Khieu et al. have found low levels of *Taenia solium* tapeworm infection in Cambodia: 0.4% in Preah Vihear province, 0.4% in children in Kandal province, and only 0.1% in Takeo province [51–53]. National estimates of infection prevalence among school children in neighboring Lao PDR were similarly low: 0–1.8% at the provincial level by stool assay [54]. The relatively low prevalence of intestinal tapeworm infection and the low prevalence of antibodies to the cysticercosis antigen in our study of adult women suggest that the risk of eliciting neurocysticercosis through mass drug administration with either praziquantel (for schistosomiasis) or albendazole (for soil transmitted helminthiasis) is likely to be low in this setting- a useful observation for the Ministry of Health in planning NTD interventions.

A somewhat surprising result from our study was the high seroprevalence of *S*. *stercoralis* infection throughout Cambodia. *S*. *stercoralis* is thought to establish life-long infection because of its propensity for autoinfection [55], and, in immunocompromised patients, a hyperinfection syndrome with a high case mortality may result [56]. The sensitivity of the *S*. *stercoralis* assay determined using samples from stool-confirmed cases suggested that the assay that was only 84% sensitive. Thus, it is possible that our results are, in fact, an underestimate of true infection prevalence. Previous surveys have documented a high prevalence (21–44.7%) of strongyloidiasis using stool assays [51–53]; the present results establish that this problem is national in scope. Strongyloidiasis does respond to ivermectin therapy and MDA with ivermectin is a cornerstone of efforts to eliminate onchocerciasis and lymphatic filariasis in sub-Saharan Africa; however, there is currently no WHO guidance on either the dosage or treatment schedule that would be required to carry out MDA with ivermectin to control *Strongyloides* in other settings. In addition, donation programs for this drug are currently restricted to the two filarial infections.

A key factor in the successful completion of this integrated survey was the forward-looking decision of survey planners to include specific language in the consent form that permitted testing for multiple infections. Such permissive language is not currently a standard feature of most surveys, and obtaining ethical approvals for retrospective testing of stored specimens can be problematic. When serum or blood spot collection is included in population-based surveys, survey planners should include permissive language in consent forms to allow a broader approach to integrated serosurveys.

Although multiplexing technology has tremendous potential for integrated serosurveys, some limitations in our study must be acknowledged. First, defining robust cutoffs to determine seropositivity can be challenging for some antigens, especially when banks of true negative sera and of positive sera from parasitologically confirmed cases are not readily available. For most antigens in our MBA panel, we used a non-endemic negative control sample set in order to establish a cutoff. This approach may not have been ideal, and additional efforts will be needed to standardize procedures and cutoff values across multiple labs.

Second, while we included in our MBA panel only highly purified recombinant antigens that had been successfully used in other serologic assay formats, sensitivity and specificity are a potential concern, especially when only one parasite antigen is used in the multiplex. Based on the earlier work of Bousema et al. [57], the antibody responses to the two *Plasmodium* spp. MSP1_19_ proteins are not expected to be cross-reactive, but we are currently examining this in more detail. We have, however, observed that the distribution of responses to helminth antigens, in particular, may be influenced by the background exposures to other helminth parasites and cross-reactivities may result in false-positives in the Bm14 response [20]. Such specificity concerns can be mitigated by including multiple parasite antigens in the MBA, as we did here with three unrelated LF antigens.

Third, the current survey only included women of child-bearing age and was specifically designed to provide seroprevalence estimates for tetanus, and rubella at the regional level [6, 7]. No epidemiologic information relative to parasitic diseases (i.e., bed net use) was collected. While the survey was well-suited for the concurrent analysis of congenital toxoplasmosis risk, the possibility of gender- or age-specific differences in either the prevalence or distribution of some of the other infections of interest must be acknowledged. For example, several studies have shown that men have a higher prevalence of strongyloidiasis than women [51, 52], and gender differences in malaria prevalence are often noted in Cambodia because men are more frequently exposed to vector mosquitoes while working in sylvan environments [58, 59]. Similarly, the study design did not take into account potential seasonal differences (important for malaria) as samples were collected only in November and December of 2012 at the beginning of the dry season [40].

Fourth, because of their small populations and remoteness, the provinces with the highest expected levels of malaria and LF were represented in the national survey by few EAs. This reflects the fact that the study was powered to compare regional prevalence estimates rather than estimates at the province, district, or EA level. Nevertheless, hot spots of parasite-specific antibody responses were observed in the nationwide survey, and, once identified, these areas could certainly be targeted for more focal screening in future surveys.

Despite these limitations, the use of the antibody multiplex assay in the context of a nationally representative survey provides a proof of principle of the potential utility of integrated programmatic monitoring and evaluation for many diseases. The multiplex assay is a flexible platform that can integrate monitoring and evaluation opportunities for various conditions and that can easily be adapted to meet country needs. It is our hope that this work will help further the idea of combining efforts for integrated monitoring and surveillance activities among global public health organizations.

## Supporting Information

S1 Fig Median plots for *P*. *falciparum* MSP1_42_ 3D7 and FVO antibody responses. (TIF)

S1 Table Weighted national estimates for toxoplasmosis and cysticercosis. (DOCX)

S1 Data Supporting information data file. (XLS)

S1 Checklist STROBE Checklist. (DOC)
