# Supplementary figures and images for: Integration of Multiplex Bead Assays for Parasitic Diseases into a National, Population-Based Serosurvey of Women 15-39 Years of Age in Cambodia
Source: PLoS Negl Trop Dis. 2016 May 3;10(5):e0004699. doi: 10.1371/journal.pntd.0004699 (PMC4854427; doi:10.1371/journal.pntd.0004699)

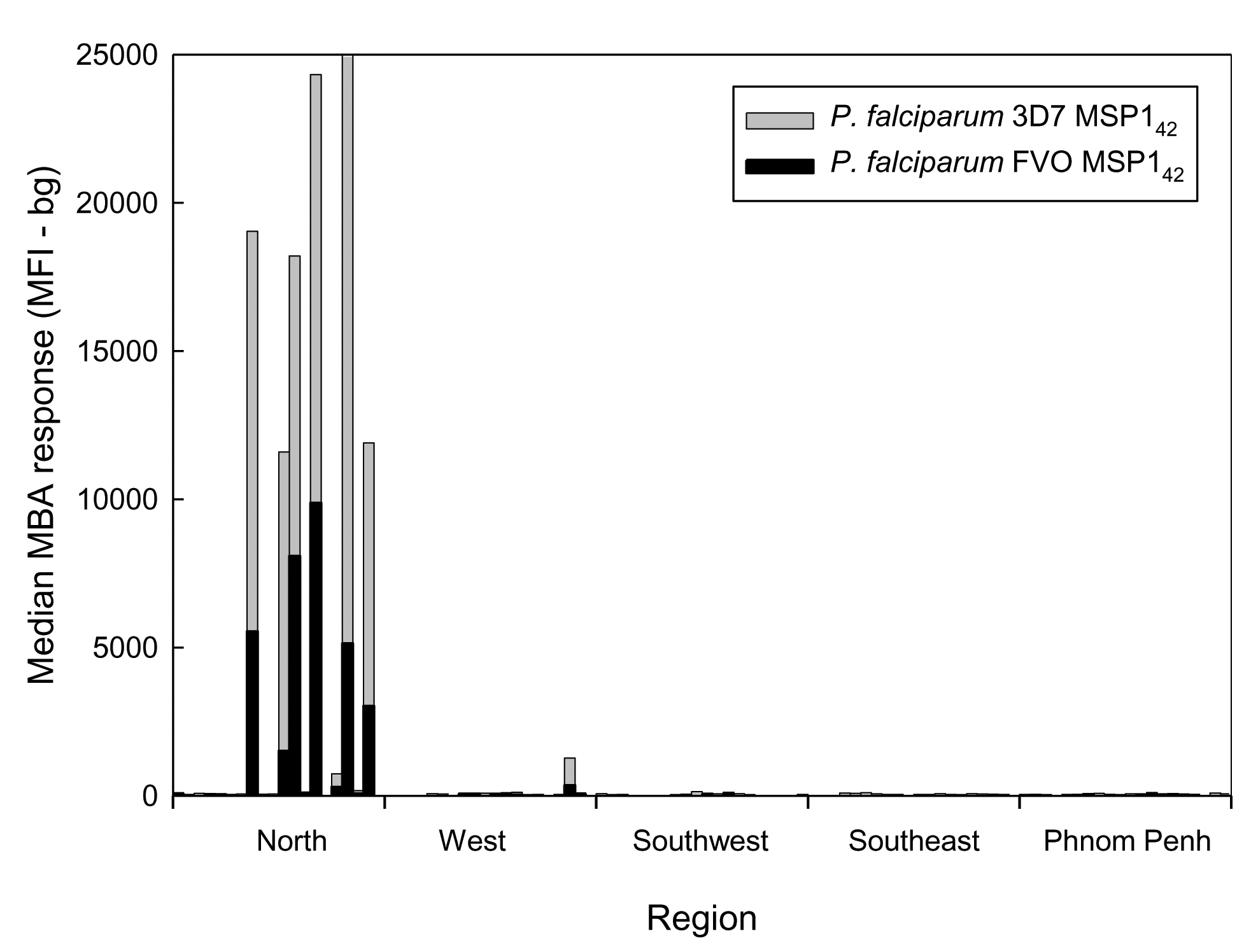

Supplement: S1 Fig — (TIF) [file pntd.0004699.s001.tif]
