# Supplementary material for: Integration of Multiplex Bead Assays for Parasitic Diseases into a National, Population-Based Serosurvey of Women 15-39 Years of Age in Cambodia
Source: PLoS Negl Trop Dis. 2016 May 3;10(5):e0004699. doi: 10.1371/journal.pntd.0004699 (PMC4854427; doi:10.1371/journal.pntd.0004699)
Supplement: S1 Table — (DOCX) [file pntd.0004699.s002.docx]

Supplemental Table 1

|  |  | ***T. gondii* SAG2A MBA*^a^*** | | | | |  | **Cysticercosis T24H MBA*^a^*** | | | | |
| --- | --- | --- | --- | --- | --- | --- | --- | --- | --- | --- | --- | --- |
| Characteristic | Total | Positive | Percent | LCL | UCL | *P* value |  | Positive | Percent | LCL | UCL | *P* value |
| Overall | 2150 | 128 | 5.8 | 4.7 | 7.0 |  |  | 60 | 2.6 | 1.8 | 3.7 |  |
| Residence type |  |  |  |  |  |  |  |  |  |  |  |  |
| Urban | 655 | 35 | 5.0 | 3.9 | 6.5 | 0.319 |  | 11 | 2.0 | 0.8 | 4.5 | 0.430 |
| Rural | 1495 | 93 | 6.0 | 4.7 | 7.6 |  |  | 49 | 2.8 | 1.9 | 4.1 |  |
| Region |  |  |  |  |  |  |  |  |  |  |  |  |
| North | 394 | 33 | 6.6 | 4.6 | 9.3 | 0.160 |  | 15 | 2.2 | 1.0 | 5.1 | 0.734 |
| West | 445 | 28 | 6.6 | 4.7 | 9.1 |  |  | 15 | 3.3 | 1.7 | 6.3 |  |
| Southwest | 423 | 30 | 7.3 | 4.2 | 12.6 |  |  | 10 | 2.6 | 1.0 | 6.4 |  |
| Southeast | 419 | 13 | 3.5 | 1.9 | 6.6 |  |  | 13 | 2.9 | 1.5 | 5.8 |  |
| Phnom Penh | 469 | 24 | 4.6 | 2.8 | 7.3 |  |  | 7 | 1.3 | 0.5 | 3.2 |  |
| Age group (yr) |  |  |  |  |  |  |  |  |  |  |  |  |
| 15-19 | 435 | 23 | 6.5 | 3.7 | 11.0 | 0.363 |  | 13 | 2.0 | 1.0 | 4.0 | 0.950 |
| 20-24 | 468 | 26 | 4.4 | 2.8 | 6.9 |  |  | 10 | 2.5 | 1.2 | 5.4 |  |
| 25-29 | 483 | 23 | 4.1 | 2.5 | 6.6 |  |  | 15 | 2.8 | 1.6 | 4.8 |  |
| 30-34 | 449 | 31 | 6.3 | 4.0 | 9.8 |  |  | 12 | 2.8 | 1.4 | 5.4 |  |
| 35-39 | 315 | 25 | 8.3 | 4.2 | 15.6 |  |  | 10 | 2.8 | 1.3 | 5.8 |  |

^a^Estimates adjusted to account for sampling weights and survey design. Abbreviations: LCL, Lower confidence limit; UCL, Upper confidence limit.
